# Navigation-Guided Trans-glenoid Flexible Fixation Technique for Arthroscopic Autologous Iliac Crest Grafting Treatment of Recurrent Shoulder Dislocation

**DOI:** 10.1016/j.eats.2022.07.017

**Published:** 2022-10-20

**Authors:** Jiaxing Huang, Li Wei, Bo Zhu, Jiawei Wang, Wei Huang, Ning Hu, Hong Chen

**Affiliations:** a Department of Orthopedics, The First Affiliated Hospital of Chongqing Medical University, Chongqing, China; b Orthopedic Laboratory, Chongqing Medical University, Chongqing, China; c Department of Orthopedics, Chengdu First People’s Hospital, Chengdu, China

## Abstract

Recurrent anterior shoulder dislocations accompanied by severe glenoid bone defects are typically treated with arthroscopy. Until now, autologous iliac grafting has been reported with excellent results, and different techniques of bone fixation have been introduced by numerous scholars. In this article, we introduce a specially designed guide that can achieve accurate positioning of the bone graft and a nonrigid graft fixation technique with a single EndoButton (Smith & Nephew). Using this technique, we greatly simplify the arthroscopic procedure and avoid the use of screws.

Recurrent shoulder dislocation after initial traumatic dislocation is fairly common owing to the relative lack of bony limitations. Moreover, extensive range of motion,^1^ young age, and high activity level serve as very strong risk factors for recurrent shoulder dislocations.^2^ Numerous surgical techniques have been described over the past 10 decades to correct this situation.^3^ For patients with extensive glenoid bone defects, bone augmentation greatly reduces the shoulder dislocation recurrence rate as compared with Bankart repair alone.^4^^,^^5^

At present, the Latarjet procedure and iliac crest grafting are the most commonly used procedures for restoration of the glenoid’s pear shape.^6^^,^^7^ The Latarjet procedure works via a mechanical barrier of a coracoid bone block and the sling effect of the conjoint tendon.^3^ However, nonanatomic reconstruction destroys the normal kinematics of the subscapularis tendon.^8^ Recently, free bone block (FBB) procedures have been proposed as an alternative to or bailout for the Latarjet procedure for the treatment of anterior shoulder instability. Moreover, it was confirmed that the safety and efficacy of the FBB procedure are fairly equal to those of the Latarjet procedure.^9^^,^^10^ Furthermore, a recent large-sample meta-analysis has shown that the rates of recurrent instability, osteoarthritis progression, and return to sports were similar between the 2 procedures.^11^^,^^12^ In addition, the aforementioned study revealed that screw fixation was related to some complications in both the Latarjet and FBB procedures^13^ (including screw loosening, screw impingement, bone nonunion, infection, poor bony position, bony resorption, and osteoarthritis) and was required highly for screw placement.^14^

Therefore, we designed a specific guide to create an accurate trans-glenoid tunnel and presents a nonrigid autologous iliac crest grafting technique, using EndoButton (Smith & Nephew) fixation, to manage recurrent shoulder dislocation.

## Preoperative Assessment

**Fig 1:**
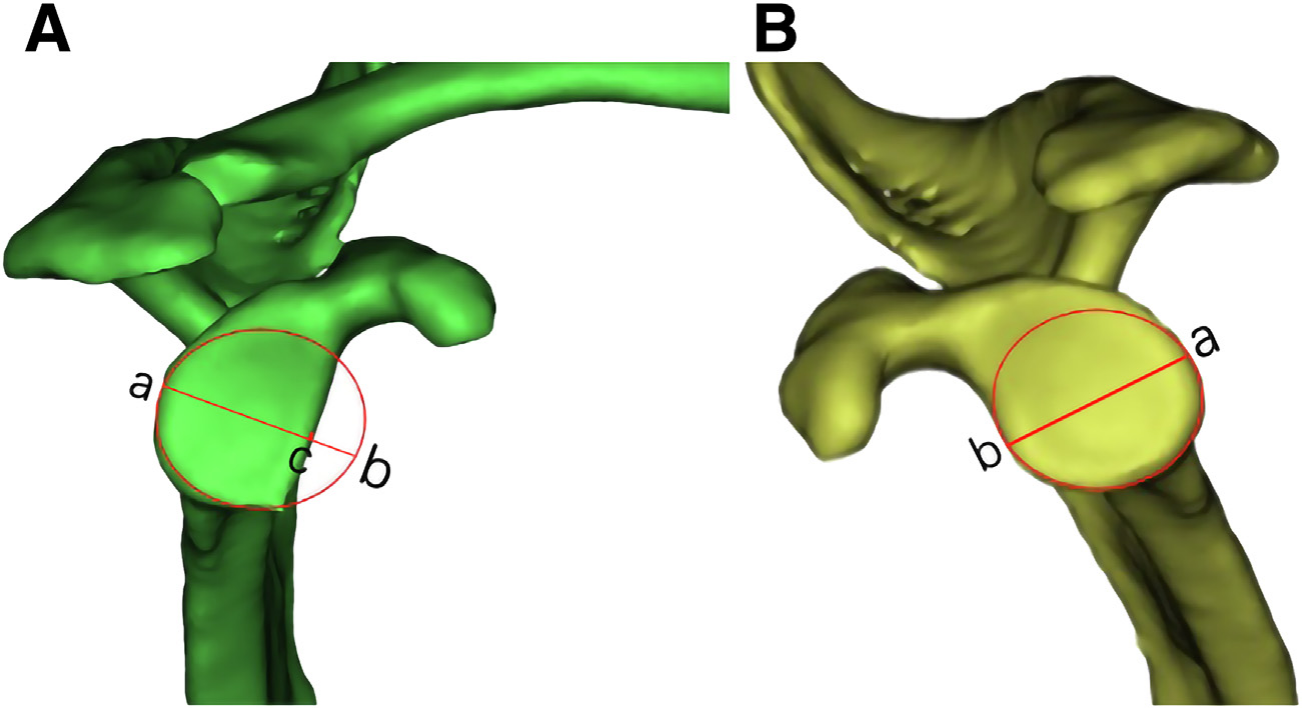
(A, B) Three-dimensional reconstruction images of the bilateral glenoid are reconstructed by Mimics software (Materialise). A virtual circle (red circle) is drawn on the normal glenoid as a reference. The same-sized circle (diameter ab) on the contralateral glenoid shows the extent of the bone defect (longest diameter [i.e., diameter bc]), and the percentage of the bone defect is roughly estimated as bc/ab.

The patients with recurrent shoulder dislocation. Testing the instability, apprehension, relocation, and load-and-shift tests. Bilateral 3-dimensional reconstruction computed tomography (CT) scans of the scapular glenoid and magnetic resonance imaging scans are routinely performed prior to the operation. We remodel the inferior aspect of the intact glenoid as a true circle, and the percentage of glenoid bone loss is calculated perioperatively. The graft size is customized, based on the individual patient, as shown in Figure 1.

## Posterior Drill Guide

**Fig 2:**
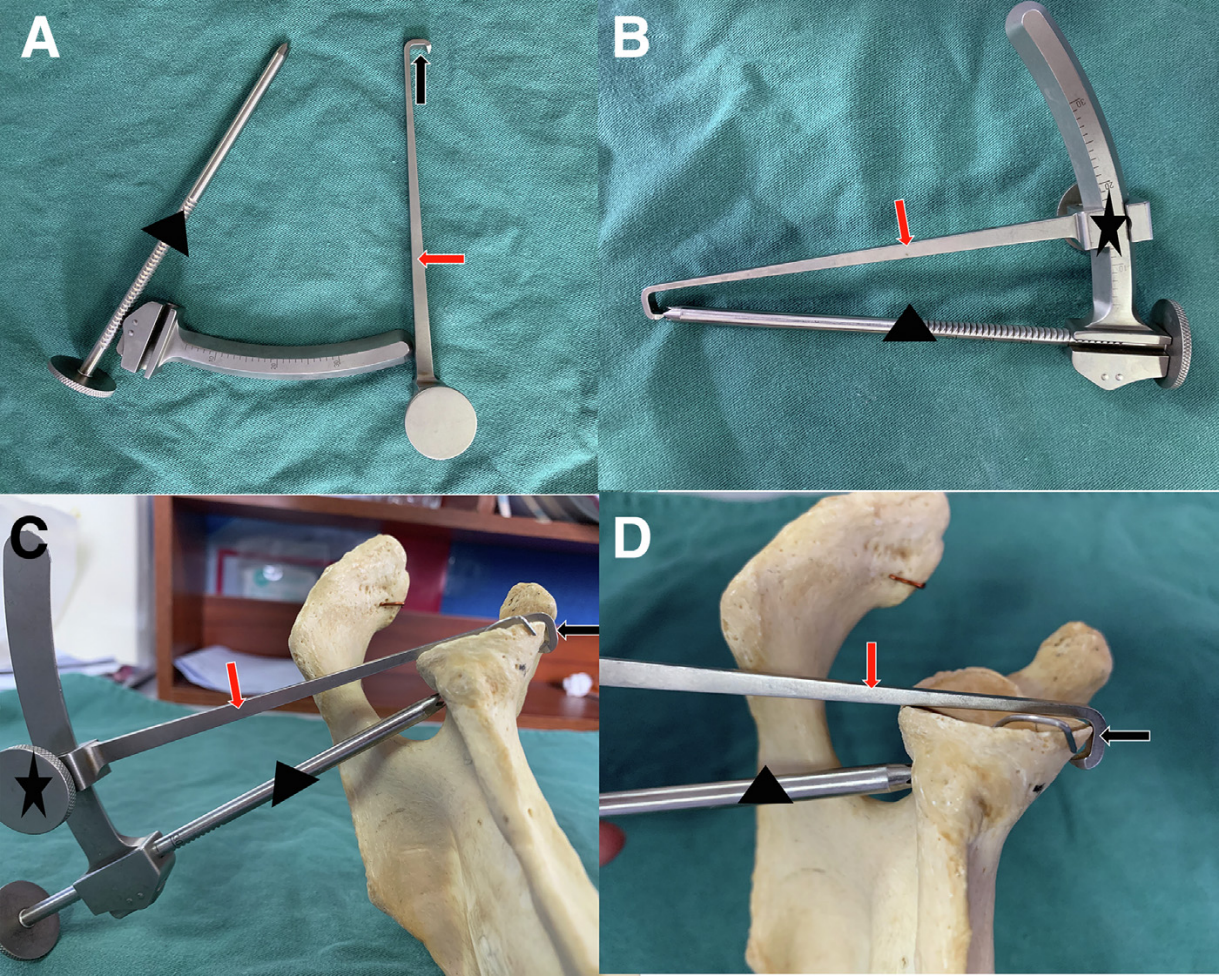
(A-D) We designed a set of bone tunnel guide instruments with 3 components. The targeting component (red arrows) with a 7-mm offset (black arrows) is introduced into the joint from the posterior portal. The hollow guiding component (black triangles) allows a 2.0-mm K-wire and keeps aligned with the anterior targeting hook. The slide component (black stars) allows the angle to be adjusted between 10° and 30° to meet different surgical needs.

The guide used in our technique is not yet commercially available and is custom manufactured by Rejoin (Hangzhou, China). This guide has 3 components (Fig 2): The hook component provides a 7-mm offset, and the remaining 2 components guide the 2.0-mm-diameter K-wire. The angle between the bone tunnel and the glenoid articular surface is adjusted between 10° and 30° to avoid injury to the suprascapular nerve.

## Surgical Technique

### Patient Positioning

**Fig 3:**
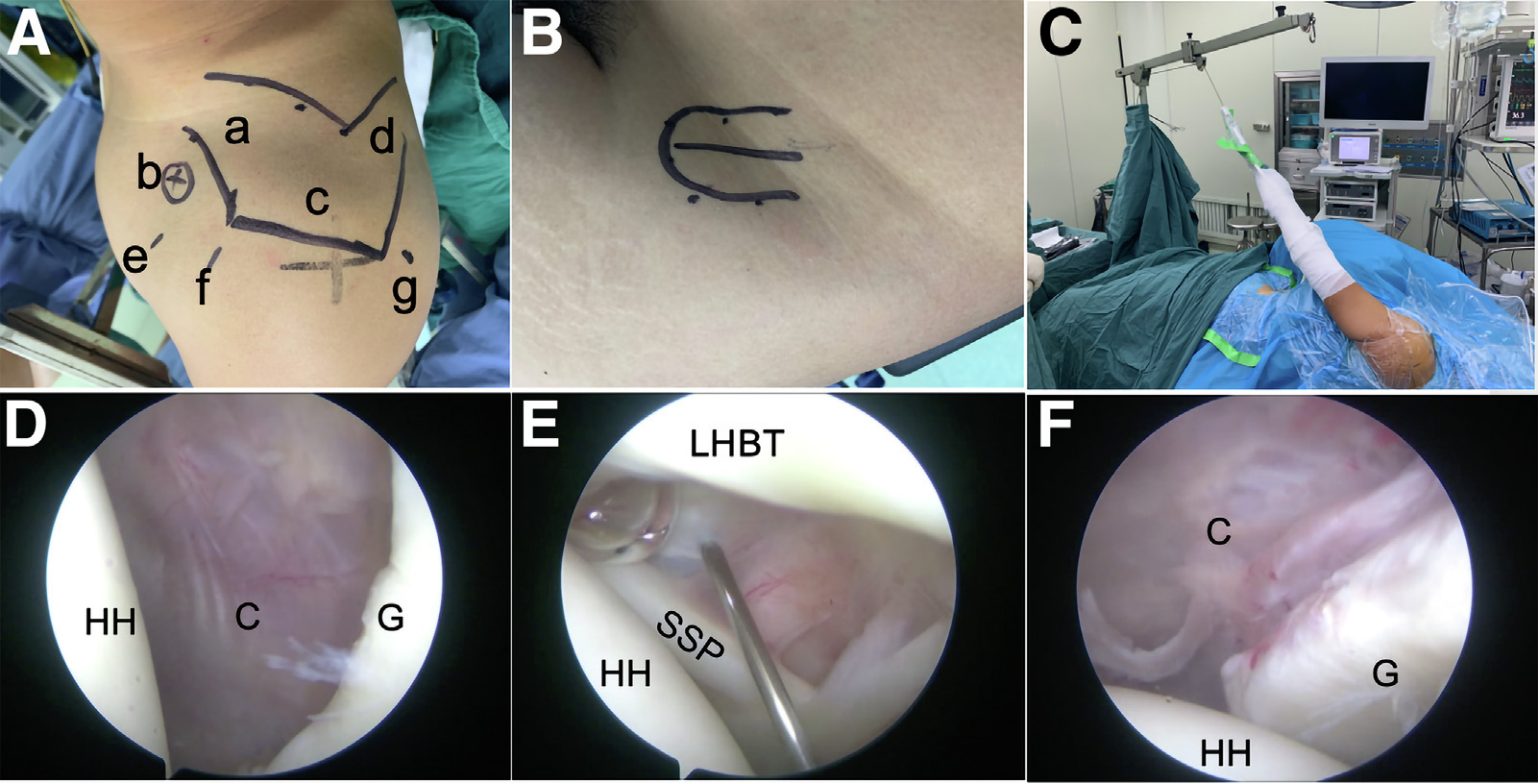
(A) Bony markers on left shoulder, including clavicle (a), coracoid (b), acromion (c), and scapular spine (d), as well as arthroscopic anterosuperior (e), anteroinferior (f), and posterior (g) portals. (B) Five-centimeter skin incision marker on anterior superior iliac spine. (C) The surgical procedure is performed with the patient in the “lazy” lateral decubitus position. (D) Diagnostic arthroscopic examination is performed from the posterior portal to view the glenoid bone defect (left shoulder, 30° arthroscope, lateral decubitus position). (E) Anterior portals are established using a needle in an outside-in manner. (F) The arthroscope is transferred to the anterior portal to observe the glenoid defect and Bankart lesion. (C, capsule; G, glenoid; HH, humeral head; LHBT, long head of biceps tendon; SSP, subscapularis.)

With the patient under general anesthesia and an interscalene block, the bony markers of the shoulder and iliac crest are drawn as shown in Figure 3 A and B. The procedure is performed with the patient in the “lazy” lateral decubitus position (Fig 3C). The patient’s body is rotated at approximately 30° to allow the surface of the glenoid to be parallel to the floor. Five kilograms of traction is maintained to keep the shoulder in 60° of flexion (to relax the anterior deltoid) and 30° of internal rotation (to enhance the space under the coracoid process while the axillary nerve is relaxed).

### Diagnostic Arthroscopy

Arthroscopy is performed via a standard posterior portal for direct visualization of the anterior glenoid bony defect (Fig 3D). The anterosuperior and anteroinferior portals are established via an outside-in technique (Fig 3E); the arthroscope is then transferred to the anterior portal for evaluation of the glenoid bone defect (Fig 3F).

### Glenoid Preparation

A shaver is used to remove any adhesions and simultaneously release the labrum-capsule from the 3- to 7-o’clock position of the glenoid to visualize the bone defect. Then, an arthroscopic burr is used to debride and decorticate the glenoid to create a flat, bleeding bony surface to accommodate the graft.

### Graft Harvest and Preparation

**Fig 4:**
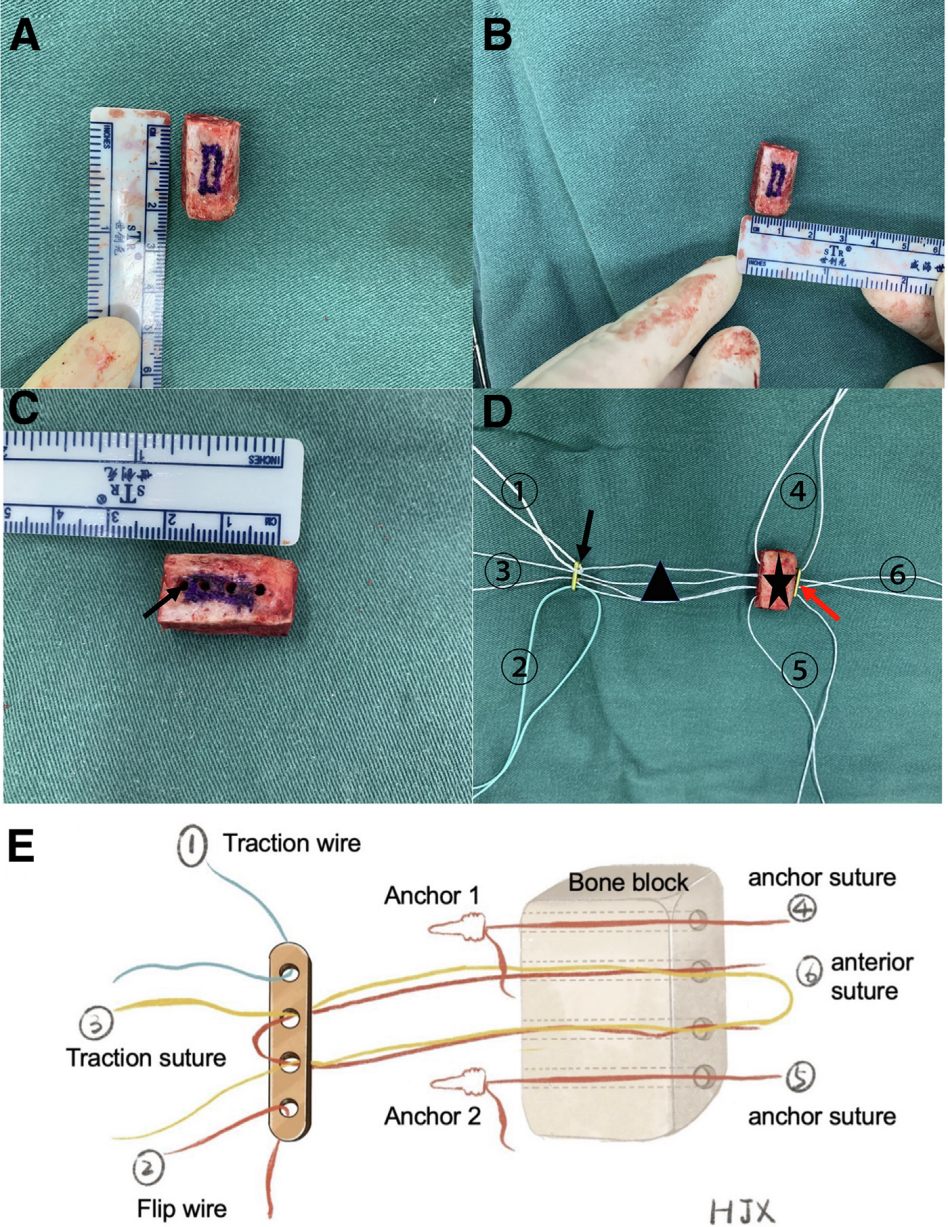
(A, B) A 20 × 10 × 10–mm tricortical iliac crest graft is harvested from the anterior superior iliac spine on the ipsilateral side per the preoperative plan. (C) Four 2-mm-wide holes are drilled by an electric drill, approximately 5 mm apart, through the graft (black arrow). (D, E) Method by which bone block (black star) is bridged with EndoButton (black arrow) using high-strength sutures (black triangle). If the patient has significant osteoporosis of the graft, an additional EndoButton (red arrow) may be added anterior to the bone block to reduce the cutting effect of the sutures leading to fracture. As shown, sutures 1, 2, and 3 are used to pull the graft anteriorly to posteriorly; of these sutures, sutures 1 and 2 are used to achieve a seesaw effect on the plate whereas suture 3 is used to tighten the bone posteriorly. Anterior sutures 4 and 5 are used as “guide sutures” to adjust the rotation of the bone block and to direct the anchors’ sutures passing through the upper and lower holes of the graft. Suture 6 is knotted in front to compress the graft.

An approximately 5-cm skin incision is made along the anterior iliac crest. A sterile ruler is used to mark the required size of the bone block, according to the preoperative plan. Any muscle and periosteum are removed from the graft, and the bone block is reshaped into a rectangular form (Fig 4 A and B). Four holes, 2 mm apart, are drilled (Fig 4C), and a high-strength suture is passed through the 2 middle holes of an EndoButton and the bone block from anterior to posterior; this is performed in the reverse direction for another suture. The latter acts as a traction wire to facilitate the introduction of the bone graft onto the glenoid neck. The 2 anchor sutures through the upper and lower holes of the bone block serve as anti-rotation wires (Fig 4 D and E).

### Glenoid Bony Channel Preparation

**Fig 5:**
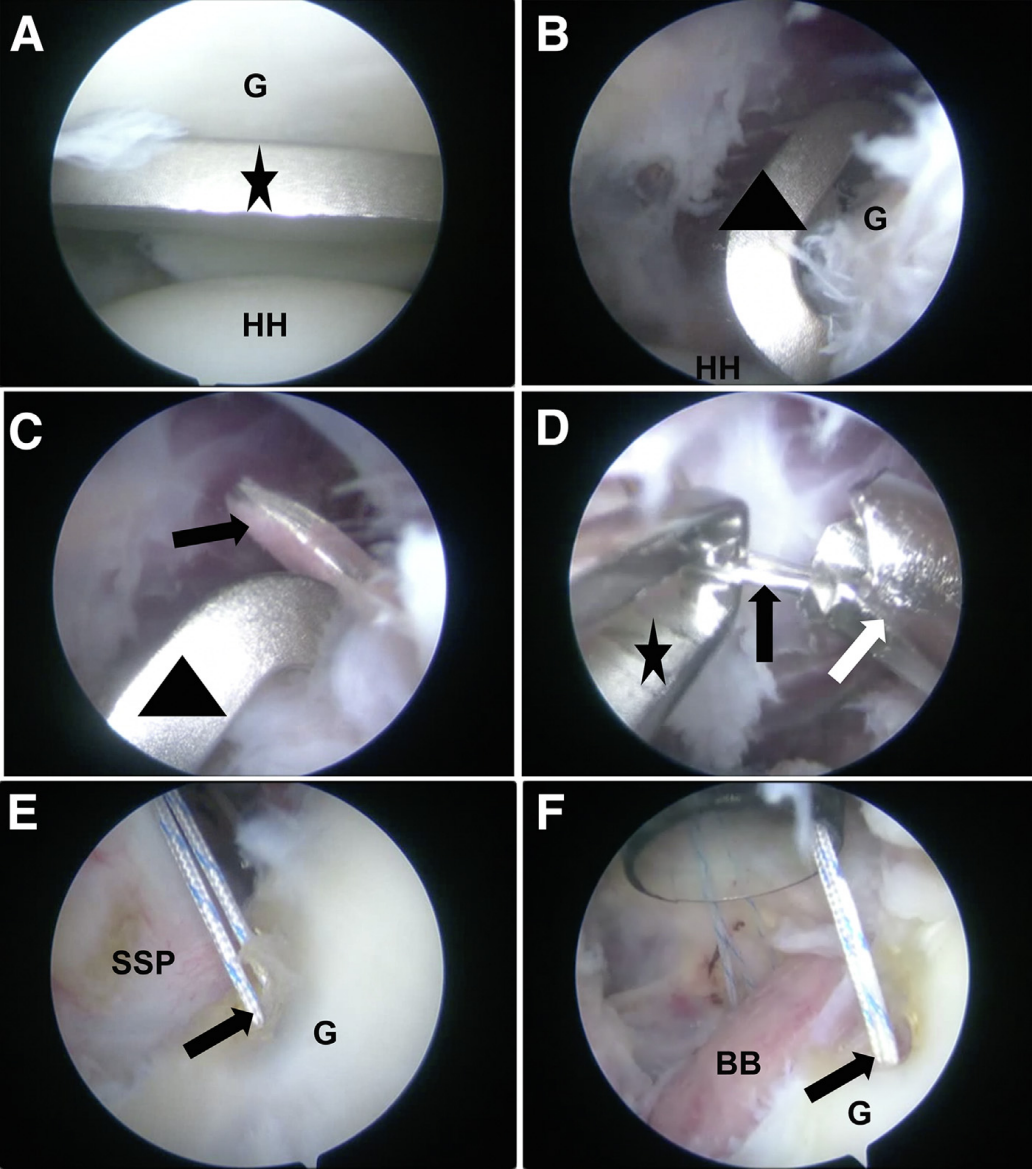
(A) The guide (black star) is introduced through the posterior portal and kept parallel to the cartilage surface to avoid injury (left shoulder, 30° arthroscope, lateral decubitus position, view from anteroinferior portal). (B) The hook (black triangle) is positioned at about the 4-o’clock position of the glenoid. (C) A 2.0-mm K-wire (black arrow) is drilled from the posterior using an electric drill following the guide access; alignment is maintained with the anterior hook (black triangle), and drilling too deeply should be avoided to prevent damage to the vessels and nerves. (D) A 4.5-mm hollow drill (white arrow) is used to enlarge the bone tunnel along the K-wire guide, the K-wire is withdrawn, a metal traction wire (black arrow) is fed through, and a grasper (black star) is introduced from the anteroinferior portal to take the wire outside (left shoulder, 30° arthroscope, lateral decubitus position, view from anterosuperior portal). (E, F) Two anchors (black arrows) are introduced at the 3- and 5-o’clock positions of the glenoid to serve as the anti-rotation suture and to perform Bankart repair, respectively (left shoulder, 30° arthroscope, lateral decubitus position, view from posterior portal). Alternatively, this step can be finished after the graft is introduced. (BB, bone block; G, glenoid; HH, humeral head; SSP, subscapularis.)

Next, the arthroscope is moved to the anteroinferior portal, and the aforementioned glenoid guide is inserted from the posterior portal. Then, we introduce the guide into the glenohumeral joint and keep it parallel to the glenoid face to avoid damaging the articular surface (Fig 5A). With its tip held anchored at the 4-o’clock position (Fig 5B), a 2.0-mm K-wire is drilled from 5 mm below the cartilage (Fig 5C); then, a 4.5-mm drill (Smith & Nephew) is used to enlarge the tunnel (Fig 5D). Subsequently, two 2.3-mm suture anchors (Smith & Nephew) are introduced at the 3- and 5-o’clock positions as landmarks for graft positioning; the anchor sutures prevent graft rotation and are used for future suturing of the anteroinferior capsule (Fig 5 E and F).

### Graft Fixation and Bankart Repair

**Fig 6:**
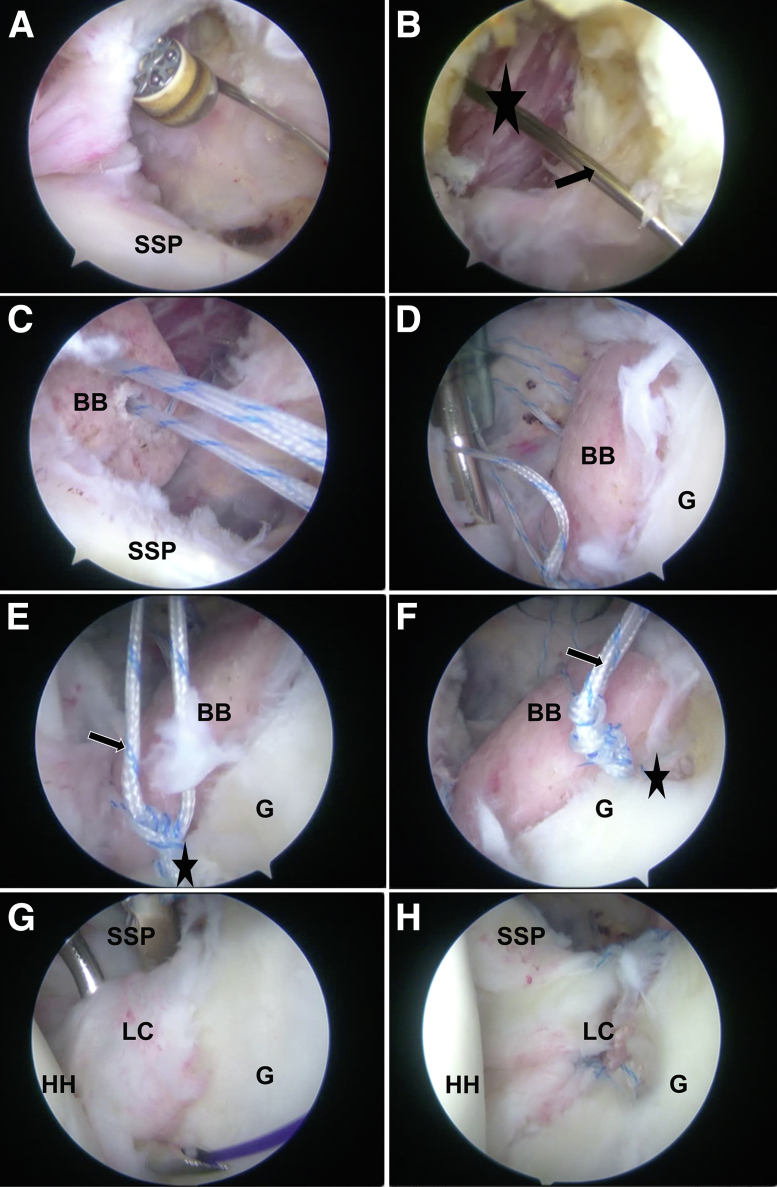
(A, B) The rotator cuff interval (black star) is cleaned sufficiently using arthroscopic tools, with removal of the access cannula of the anteroinferior portal and enlargement of the incision to ensure that the graft can be introduced smoothly through the guidewire (black arrow) (left shoulder, 30° arthroscope, lateral decubitus position, view from posterior portal). The bone block is introduced into the joint through the rotator cuff interval and undergoes traction posteriorly. (C) Tension is maintained on the anterior sutures (suture 6 in Fig 4E) as the EndoButton passes over the bone tunnel and a seesaw sensation occurs (left shoulder, viewed from above). The grasper is introduced from the anterior 2 portals to assist in proper placement and to ensure there is no rotation and lateralization of the graft and it fits well to the bone defect surface. (D) After the final adjustment is completed, tension is maintained on the posterior sutures (suture 3 in Fig 4E) (left shoulder, viewed from above). (E, F) The 2 anchors’ sutures (black stars) are passed through the bone holes following the guide sutures (sutures 4 and 5 in Fig 4D), and the tails of the sutures (black arrows) are retained after knotting (left shoulder, viewed from above). (G) The capsule-labrum structure is pulled over the graft and tightened using the remaining suture (black arrow) of the 2 anchors (left shoulder, viewed from above). (H) The capsule-labrum repair is completed; the bone graft is placed flush with the cartilage surface and kept outside the joint capsule (left shoulder, viewed from above). (BB, bone block; G, glenoid; HH, humeral head; LC, labrum-capsule; SSP, subscapularis.)

The rotator cuff interval is fully cleared (Fig 6A), and the bone graft is then introduced into the joint space with the help of traction wires (Fig 6 B and C). We next posteriorly pull the traction and flip sutures while keeping the anterior sutures under tension until a “seesaw” sensation is achieved. A grasper from the anterior portal is used to assist in adjusting the graft to achieve proper placement. After the final adjustments (Fig 6D), the anterior anti-rotation anchor sutures are knotted (Fig 6 E and F), and the posterior traction suture is tightened and knotted; finally, the anterior suture is knotted. The 2 anchor sutures tails are not cut and are used for labrum-capsule repair, and the additional anchor will be introduced if needed to keep the graft outside the joint (Fig 6 G and H). Finally, all arthroscopic instruments are removed, and the skin incisions are closed and sterilely dressed. Video 1, including narration, may provide a better understanding of our entire surgical technique.

### Postoperative Rehabilitation

The patient is discharged 1 day after the operation. A CT scan is used to evaluate the positioning of the graft and plate. The affected shoulder is immobilized with a neutral-rotation sling for 6 weeks. Elbow and wrist exercises are allowed immediately after the operation. Passive shoulder activity training is started at 3 weeks postoperatively, and full range of motion is allowed at 6 weeks postoperatively. However, heavy lifting is prohibited for at least 12 weeks postoperatively, until the graft has achieved full bony healing.

### Radiographic Assessments

**Fig 7:**
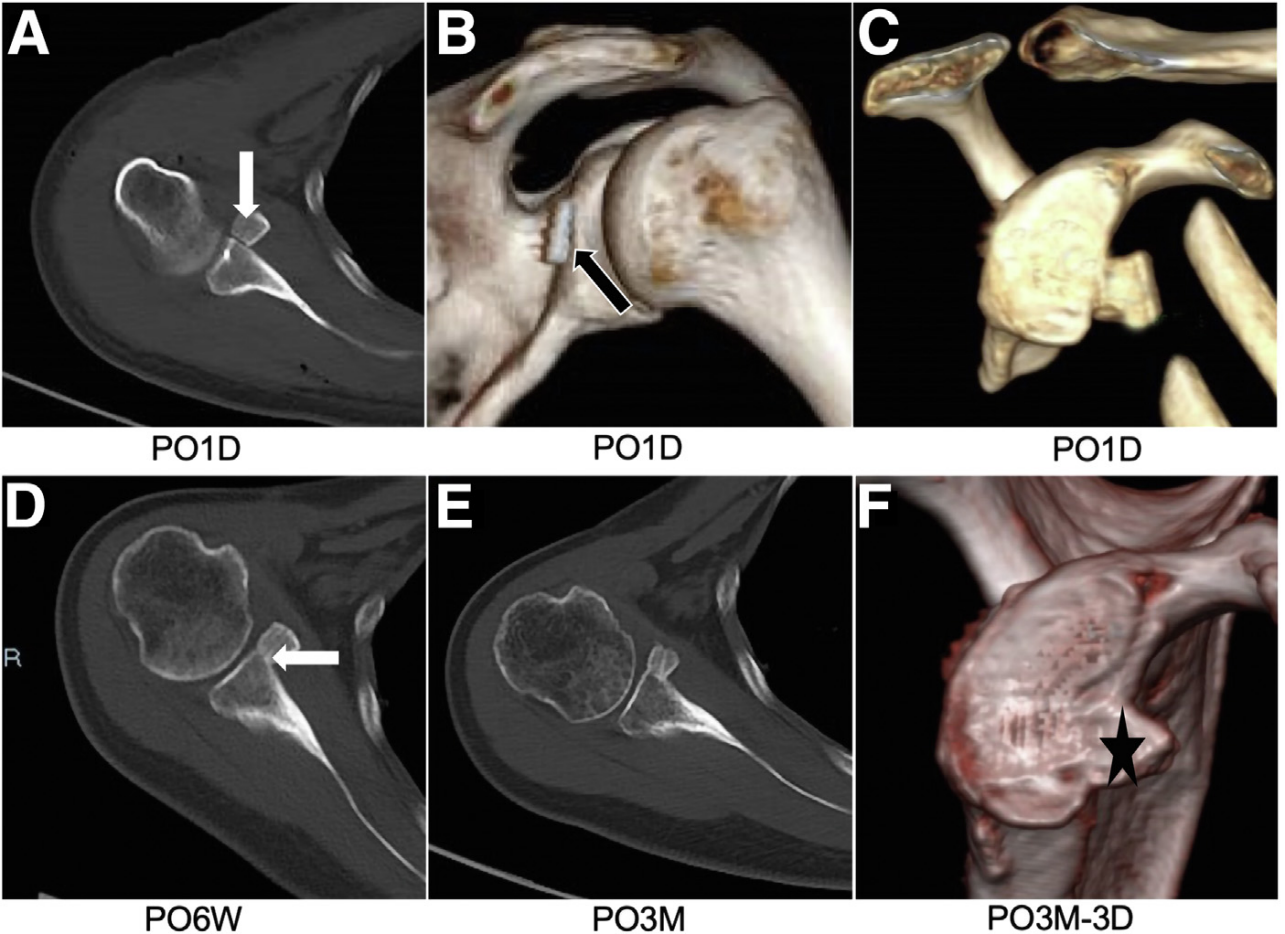
Computed tomography (CT) images of right (R) shoulders in typical patients at postoperative follow-up. (A-C) CT images on first day postoperatively (PO1D) showing good positioning of bone block (white arrow) and posterior EndoButton (black arrow). (D) CT scan at 6 weeks postoperatively (PO6W) showing good bone healing (white arrow). (E, F) Complete bone healing and remodeling of glenoid (black star) at 3 months postoperatively (PO3M). PO3M-3D, post-operatively 3 months-3 dimensional reconstruction.

As part of our routine imaging evaluation, a 3-dimensional CT scan is performed at 6 weeks and 6 months postoperatively to assess graft positioning and bone healing. The ideal graft positioning is defined as below the glenoid equator (in the vertical plane) and flush against the glenoid rim (in the horizontal plane),^15^ as shown in Figure 7.

## Discussion

A prior study revealed that 90% of patients with post-traumatic recurrent shoulder dislocation exhibit significant glenoid bone loss.^16^ Generally, 19% to 30% of glenoid defects necessitate bone surgery.^17^, ^18^, ^19^ A recent finite element analysis revealed that bone grafting, not soft-tissue repair alone, must be considered if the bone defect exceeds 16%. Otherwise, stability may not be achieved.^20^ Xiang et al.^21^ reported that autologous scapular spine bone grafts provide satisfactory results in patients with subcritical (10%-15%) glenoid bone loss.

There are 2 major approaches to address glenoid bone defects. One involves the classic Bristow-Latarjet (coracoid transposition) procedure, which uses the sling effect of the coracoid and conjoined tendon and gains better anterior-inferior stability as compared with other free bone graft procedures. However, splitting the subscapularis tendon produces long-term internal rotation forces and endurance deficits relative to the healthy shoulder.^22^, ^23^, ^24^ Moreover, it requires a special guide system for positioning and fixation of the graft and has a steep learning curve, which generally limits its clinical application.^25^

Glenoid bone grafting procedures are another major category of treatment that corrects glenoid defects. Mochizuki et al.^26^ first reported a procedure in which the graft was transplanted from the lateral side of the acromion to the glenoid defect area. However, the graft in this procedure was too small to restore the glenoid defect. In a subsequent study, a glenoid-labrum allograft was used by Skendzel and Sekiya^27^ to correct glenoid defects. In their procedure, they repaired the native glenoid and sutured the capsule-labrum structure to the labrum allograft. In our technique, the bone block is sufficient to restore the area of the glenoid bone defect. We also introduce the graft through the rotator cuff interval, without affecting the subscapularis tendon. Finally, we suture the capsule-labrum tissue to the native edge of the articular cartilage, which has allowed us to establish an enhanced healing rate owing to the native-to-native reattachment. In addition, according to the theory of Zhao et al.,^28^ the buttressing effect of the structures surrounding the graft, rather than the rigid fixation itself, plays a critical function in bone healing. Notably, the bone block may slightly displace inward over time owing to the compression effect of the capsule–glenoid labrum tissue, even if the bone block has been poorly placed.

Graft fixation is a critical aspect of defective glenoid correction. Fixation via 2 hollow screws is the most common method used during Latarjet surgery, and when applied during some free bone graft techniques, it has been shown to achieve good biomechanical and clinical outcomes.^29^, ^30^, ^31^ However, screws are linked to several hardware complications and are a common contributor to Latarjet revision surgery.^32^^,^^33^ Therefore, flexible fixation is currently one of the most widely used modifications. Multiple studies have reported promising outcomes with nonrigid fixation, and it was confirmed as a safe and reliable alternative method of screw fixation.^33^, ^34^, ^35^ In our procedure, we adopted a single EndoButton to perform an elastic fixation, which greatly simplified the procedure. Nevertheless, an additional piece of EndoButton in front of the graft (Fig 4D, red arrow) is considered to be useful in those patients with osteoporosis to reduce the shearing force of the sutures in the bone holes to avoid graft fractures. Although elastic fixation is not sufficient to provide firm fixation, nonrigid fixation offers the possibility of micromovement of the bone graft, which is helpful to adjust to the optimal position for healing; moreover, absorption and remodeling will be better, which will more closely achieve the original pear shape. In addition, the repair of the anterior capsule-labrum complex facilitates healing and improves anterior stability.^28^

The specific guide used in this technique must be noted. With the special instruments used in our technique, the precise bone channel positioning can be drilled with notable accuracy and safety. A 5-mm offset in the hook of the guide decreases the possibility of the graft overly protruding through the glenoid articular surface, which in turn reduces the incidence of arthritis. The guide can also be used in other surgical procedures, such as modified coracoid transposition. The advantages and disadvantages of our proposed technique are listed in Table 1.

**Table 1:** Advantages and Disadvantages of Technique

Advantages
The graft is introduced via the rotator cuff interval, which avoids splitting the subscapularis tendon.
The posterior traction wire helps in achieving easy placement of the graft.
The posterior EndoButton provides reliable elastic fixation, as compared with the use of anchors alone.
The technique does not involve any metal components anterior to the glenohumeral joint.
Disadvantages
Donor-site morbidity can occur.
The technique has a relatively steep learning curve.

## Supplementary Data

ICMJE author disclosure forms

Video 1 Arthroscopic autologous iliac crest bone grafting for reconstruction of glenoid using nonrigid fixation technique in left shoulder.
